# Activation of Sirtuin 3 by Silybin Attenuates Mitochondrial Dysfunction in Cisplatin-induced Acute Kidney Injury

**DOI:** 10.3389/fphar.2017.00178

**Published:** 2017-04-05

**Authors:** Yin Li, Zengchun Ye, Weiyan Lai, Jialing Rao, Wanbing Huang, Xiaohao Zhang, Ziying Yao, Tanqi Lou

**Affiliations:** Department of Nephrology, Third Affiliated Hospital, Sun Yat-sen UniversityGuangzhou, China

**Keywords:** silybin, cisplatin-induced acute kidney disease, mitochondria, SIRT3, apoptosis, regeneration

## Abstract

Silybin is a secondary metabolite isolated from the seeds of blessed milk thistle (*Silybum marianum*) that has anti-inflammatory, antioxidative, antifibrotic, and antitumor properties. Here, we showed that silybin protected against cisplatin-induced acute kidney injury (AKI) by improving mitochondrial function through the regulation of sirtuin 3 (SIRT3) expression. Male SV129 and SIRT3 knockout (KO) mice were administered a single intraperitoneal (i.p.) injection of cisplatin with or without treatment with silybin. Moreover, cultured HK2 cells were used to evaluate mitochondrial morphology and function. Our data suggested that silybin enhanced SIRT3 expression after cisplatin administration both *in vivo* and *in vitro*. Silybin treatment improved mitochondrial function and bioenergetics in wild-type, but not SIRT3-defective, cells and mice. Moreover, we demonstrated that silybin markedly attenuated cisplatin-induced AKI and tubular cell apoptosis and improved cell regeneration in a SIRT3-dependent manner. Collectively, these results suggest that silybin is a pharmacological activator of SIRT3 capable of protecting against cisplatin-induced tubular cell apoptosis and AKI by improving mitochondrial function. Thus, silybin could serve as a potential clinical renoprotective adjuvant treatment in cisplatin chemotherapy.

## Introduction

Cisplatin is one of the most efficient and widely used anticancer chemotherapy drugs in the clinic for the treatment of bladder cancer, non-small cell lung cancer and other solid tumors (Siddik, 2003). However, its use is frequently limited by its adverse effects, such as neurotoxicity, bone marrow suppression and nephrotoxicity. Among these effects, nephrotoxicity is the major limiting factor for cisplatin therapy (Arany and Safirstein, 2003). The nephrotoxic effect of cisplatin is cumulative and dose-dependent, and it often necessitates dose reduction or withdrawal (Pabla and Dong, 2008).

It has been suggested that oxidative stress, a redox state imbalance, impaired energetic metabolism, cell apoptosis and inflammation are likely associated with cisplatin-induced acute kidney injury (AKI) (Miller et al., 2010). Recent studies have also demonstrated that mitochondrial dysfunction occurs in tubular epithelial cell injury and negatively affects the recovery of renal function in cisplatin-induced AKI (Zhan et al., 2013; Yang et al., 2014). Mitochondria are remarkably dynamic organelles that constantly undergo fusion and fission to maintain their integrity and quantity, and they play essential roles in many aspects of biology in tubular epithelial cells (Brooks et al., 2009; Palmer et al., 2011; Friedman and Nunnari, 2014). Sirtuin 3 (SIRT3) has been demonstrated to protect against tubular injury in a cisplatin-induced AKI animal model by preserving mitochondrial integrity, suggesting that elevating renal SIRT3 expression to improve mitochondrial dynamics is a potential strategy for improving the outcome of AKI (Morigi et al., 2015). SIRT3 is an important member of the sirtuin family, and it is the major acetylation enzyme located in mammalian mitochondria (Michishita et al., 2005). Recent studies have indicated that metabolic enzymes and oxidative phosphorylation proteins that are potentially regulated via deacetylation by SIRT3 are important for mitochondrial energy production, metabolic homeostasis, and cell survival and longevity (Ahn et al., 2008; Verdin et al., 2010; Wu et al., 2013). In addition, SIRT3 increases the activities of antioxidant enzymes and enhances the clearance of mitochondrial reactive oxygen species (mtROS), thereby reducing ROS-mediated cell damage and maintaining cellular homeostasis (Kong et al., 2010; Bell and Guarente, 2011; Chen et al., 2013). However, an effective strategy for the treatment of tubular mitochondrial dysfunction in AKI remains to be identified.

Silybin, a polyphenolic flavonoid, is widely used to treat liver diseases due to its antioxidant, anti-inflammatory and antifibrogenic properties and its ability to stimulate DNA and RNA synthesis (Loguercio and Festi, 2011). The mechanism underlying cisplatin renal toxicity has been proposed to be the generation of free oxygen radicals and the inhibition of DNA, RNA, and protein synthesis (Miller et al., 2010). Therefore, we hypothesized that silybin would have an effect on the kidney in cisplatin-induced AKI. In the present study, we found that silybin improved mitochondrial function in tubular epithelial cells and ameliorated the decline in renal function in cisplatin-induced AKI. In addition, we found that SIRT3 expression was significantly repressed in tubular epithelial cells in cisplatin induced-AKI and that the administration of silybin increased this expression, leading to improvements in mitochondrial bioenergetics and kidney function. Taken together, our results provide evidence of a direct interaction between silybin and SIRT3 protein, suggesting that silybin therapy can optimize the clinical outcomes of cisplatin induced-AKI patients.

## Materials and Methods

### Mice and AKI Model

Animal maintenance and experimental procedures were conducted in accordance with protocols approved by the Animal Care Committee of the Sun Yat-sen University. SIRT3 knockout (KO) mice in an SV129 background were purchased from the Jackson Laboratory, and wild-type (WT) control mice were acquired from the Model Animal Research Center of Nanjing University (China). Male mice weighing approximately 20–25 g were administered cisplatin (20 mg/kg; Sigma–Aldrich, USA) or a vehicle by a single i.p. injection as previous articles reported (Gao et al., 2016; Suzuki et al., 2016). To investigate the effects of silybin on cisplatin-induced AKI, the mice were pretreated with 200 mg/kg silybin per day by intragastric administration for 7 days before and 3 days after cisplatin injection. The mice were sacrificed under anesthesia 72 h after cisplatin administration, and blood and kidney samples were collected and harvested for biochemical and histopathologic examinations.

### Cell Culture

HK2 cells were obtained from the American Type Culture Collection (Manassas, VA, USA) and were grown in DMEM/F-12 (Gibco, USA) supplemented with 10% fetal bovine serum (FBS; Gibco, USA), 100 U/ml penicillin and 100 μg/ml streptomycin in a humidified atmosphere of 5% CO_2_/95% air at 37°C. SIRT3 siRNA (Ribobio, China) was transfected into HK2 cells using Lipofectamine 3000 reagent (Invitrogen, USA) according to the manufacturer’s instructions. At 48 h after transfection, the cells were stimulated with cisplatin (5 μmol/L) for an additional 24 h in the presence or absence of silybin (50 μmol/L).

### Histology and Immunohistochemistry

The kidney samples were fixed in 4% paraformaldehyde, stained with periodic acid-Schiff (PAS) staining, and observed by light microscopy. Cellular casts and necrosis were assessed in at least 20 randomly chosen fields by microscopy (×400). For SIRT3 immunohistochemical staining, 5-μm kidney sections were incubated with anti-SIRT3 (Cell Signaling Technology, USA), followed by incubation with the respective secondary antibodies. The quantification of SIRT3-positive tubular regions was performed by examining at least 20 fields (×200) for each mouse.

### Transmission Electron Microscopy

Fragments of cortical kidney tissues were fixed in 2.5% glutaraldehyde in 0.1 M cacodylate buffer for at least 2 h. Then, they were fixed in 1% osmium tetroxide, dehydrated with ascending concentrations of ethanol, subjected to resin penetration and embedded in epon. After solidification, the tissues were cut into ultrathin sections and stained with uranyl acetate, followed by staining with lead citrate; finally, they were examined using a JEM-1400 transmission electron microscope (Japan). The aspect ratio was calculated as previous published methods (Ayanga et al., 2016).

### Mitochondrial Function

Living HK2 cells were labeled with 300 nM MitoTracker Deep Red (Invitrogen, USA) for 15 min at 37°C. Mitochondrial membrane potential was measured using the fluorescent dye tetramethylrhodamine methyl ester (TMRM, 250 nM, Invitrogen, USA) for 20 min. mtROS was detected by labeling cells with MitoSOX Red (5 μM, Invitrogen) for 10 min. After incubation, the cells were washed several times and examined under an inverted confocal microscope. The images were analyzed by ImageJ for integrated density analysis. At least 100 cells were determined for each condition.

### Real-time PCR for SIRT3 and Quantification of mtDNA

Total RNA was extracted from HK2 cells and mouse renal tissues using TRIzol reagent (Invitrogen, USA) according to the manufacturer’s instructions. Then, the total RNA was reverse transcribed using a PrimeScript^TM^ RT Reagent Kit with gDNA Eraser (TaKaRa, Japan). SYBR^®^ Premix Ex Taq^TM^ II (TaKaRa, Japan) was used to amplify SIRT3 cDNA. SIRT3 gene expression was normalized by β-actin expression.

Total DNA was extracted using a MiniBEST Universal Genomic DNA Extraction Kit (TaKaRa, Japan). To determine the mtDNA/gDNA ratio, qPCR was performed to amplify the mitochondrial genome (mt16s) and nuclear genome (B2M), as previously described (Bueno et al., 2015). The expression of mtDNA was normalized by that of nuclear DNA using the comparative cycle threshold (^∆∆^ Ct) method.

The sequences of the primers used for PCR were as follows: SIRT3 (mouse): forward, CACGTTTACAAACATGAACC, reverse, CATGCTAGATTGCCCTAGT; β-actin (mouse): forward, AGACCTTCAACACCCCAG, reverse, CACGATTTCCCTCTCAGC; SIRT3 (human): forward, TGGCACCCAGCACAATGAA, reverse, CTAAGTCATAGTCCGCCTAGAAGCA; β-actin (human): forward, TGGCACCCAGCACAATGAA, reverse, CTAAGTCATAGTCCGCCTAGAAGCA; B2M (human): forward, TGCTGTCTCCATGTTTGATGTATCT, reverse, TCTCTGCTCCCCACCTCTAGGT; and mt16S (human): forward, GCCTTCCCCCGTAAATGATA, reverse, TTATGCGATTACCGGGCTCT.

### ATP Production

Cellular ATP levels were determined using an Enhanced ATP Assay Kit (Beyotime, China). Lysed cells were centrifuged for 5 min at 4°C and 12,000 *g*, and the supernatant was collected. Before ATP detection, detecting solution was added to a 96-well plate and incubated at room temperature for 5 min. The supernatant was then added to the plate, mixed quickly, and read within 30 min. Total ATP levels were calculated from the luminescence signals and were normalized by the protein concentrations.

### Apoptosis Analysis

For Annexin V FITC (BD Biosciences, San Diego, CA, USA) labeling, HK2 cells were gathered by trypsinization, washed in cold phosphate-buffered serum (PBS), and then incubated with Annexin V FITC according to the manufacturer’s instructions. Next, the cells were analyzed by flow cytometry.

Kidney apoptosis was measured using terminal deoxynucleotidyl transferase-mediated dUTP nick-end labeling (TUNEL) staining (Roche Molecular Biochemicals, Germany). The rate of apoptosis was calculated by examining at least 20 fields for each sample.

### Proliferation Assay

At 72 h after cisplatin administration, the mice were administered bromodeoxyuridine (BrdU; Sigma–Aldrich, USA) by i.p. injection at a dose of 50 mg/kg body weight, and they were sacrificed 2 h later. The proliferation of renal tubular cells was identified by immunohistochemical analysis of BrdU and proliferating cell nuclear antigen (PCNA). At least 10 fields (×200) were examined for each mouse.

### Immunoblotting

Cells were lysed in radioimmunoprecipitation assay (RIPA) buffer (Thermo Fisher, USA) containing protease inhibitors (Thermo Fisher, USA) before being scraped off and centrifuged for 15 min at 4°C and 12,000 *g*. Equal amounts of protein were loaded onto 12% SDS-PAGE gels and transferred to PVDF membranes (Millipore Corp., USA). The membranes were blocked with 5% bovine serum albumin (BSA) in TBST for 60 min and then incubated with a monoclonal rabbit anti-SIRT3 antibody (1:1000, CST) or polyclonal rabbit anti-cleaved caspase-3 antibody (1:1000, CST) with monoclonal β-actin antibody-HRP (1:10000, Bioworld) overnight at 4°C. Subsequently, the membranes were incubated with a horseradish peroxidase (HRP)-labeled goat anti-rabbit antibody (1:20000; Bioworld Technology, USA) in TBST with 5% BSA for 60 min at room temperature. The blots were visualized by enhanced chemiluminescence (ECL; Millipore, USA), and the bands were quantified using ImageJ software (National Institutes of Health, Bethesda, MD, USA).

### Statistical Analysis

All data were presented as the means ± SEM from three independent experiments. A statistical comparison of the groups was performed using the *t*-test or one-way analysis of variance (ANOVA), followed by Bonferroni’s *post hoc* test. P < 0.05 was considered to indicate statistical significance in all tests. SPSS 15.0 software was used for statistical analyses (SPSS Inc., USA).

## Results

### Silybin Protects against Cisplatin-induced AKI

**FIGURE 1:**
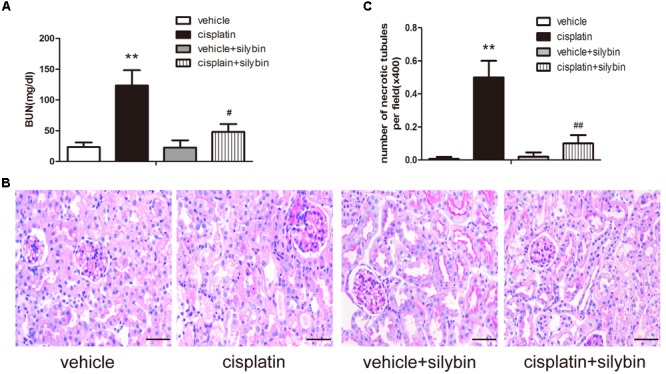
**Silybin protects against cisplatin-induced acute kidney injury (AKI).** Male SV129 mice were treated with vehicle and cisplatin with or without silybin (*n* = 6). **(A)** The graph shows the blood urea nitrogen (BUN) levels of mice 72 h after cisplatin injection. **(B)** Histological examination of periodic acid-Schiff (PAS) staining in each group and **(C)** quantitation of tubular necrosis. Scale bar, 50 μm. ^∗∗^*P* < 0.01 vs. vehicle mice; #*P* < 0.05, ##*P* < 0.01 vs. mice treated with cisplatin alone.

To assess the effects of silybin on cisplatin-induced AKI, male SV129 mice were i.p. injected with 20 mg/kg cisplatin to induce AKI. After 3 days, the mice developed AKI, as demonstrated by their elevated creatinine levels. To determine the role of silybin in cisplatin-induced AKI, the mice were pretreated with 200 mg/kg silybin by intragastric administration for 7 days before and 3 days after cisplatin injection. The blood urea nitrogen level was significantly decreased in the silybin group compared with the cisplatin group (**Figure 1A**).

Periodic acid-Schiff staining revealed apparently normal kidney tubules in the vehicle group. However, the cisplatin group exhibited severe kidney histological abnormalities, including renal tubular epithelial cell edema and detachment. However, the injury-promoting processes, including epithelial cell atrophy and necrosis, were significantly attenuated by pretreatment with silybin (**Figures 1B,C**).

Taken together, these data indicated that silybin had a protective effect on cisplatin-induced AKI compared with that observed in the mice treated with cisplatin alone.

### Effects of Silybin on Mitochondria in Cisplatin-induced AKI

**FIGURE 2:**
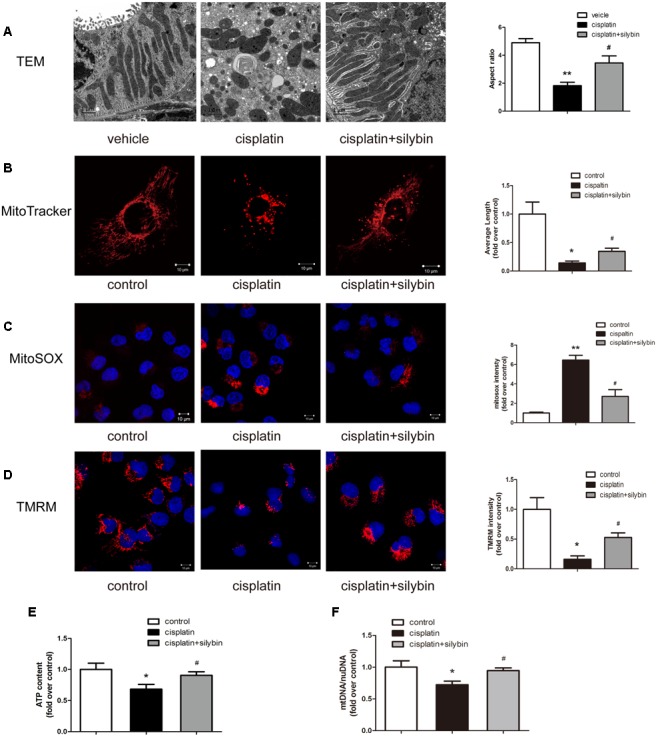
**Silybin improves mitochondria fitness. (A)** Representative TEM micrographs of mouse renal tubular epithelial cell mitochondria from each group and average aspect ratio. Scale bar, 1 μm. **(B)** Representative images showing the mitochondrial morphology of HK2 cells after staining with MitoTracker Deep Red and quantitative analysis. Scale bar, 10 μm. **(C,D)** MitoSOX **(C)**, TMRM **(D)**, and quantitative analysis were used to examine mtROS and mitochondrial membrane potential in HK2 cells. Scale bar, 10 μm. **(E)** ATP levels were quantified in HK2 cells. **(F)** Mitochondrial mass of HK2 cells was assessed using the mtDNA/nDNA ratio. The data represent the means ± SEM. ^∗^*P* < 0.05, ^∗∗^*P* < 0.01 vs. vehicle mice or control cells; #*P* < 0.05 vs. mice or cells treated with cisplatin alone.

Three days after cisplatin administration, the cortices of the mouse kidneys were observed using electron micrography to evaluate the mitochondria. The control group had elongated mitochondrial profiles; in contrast, the cisplatin group was found to have small, fragmented, swollen and abnormal mitochondrial profiles, while the silybin group had longer mitochondrial profiles and reduced mitochondrial swelling than the cisplatin group (**Figure 2A**). Consistent with the *in vivo* findings, the exposure of HK2 cells to cisplatin resulted in small and fragmented mitochondria, and treatment with silybin caused enlargement of the mitochondria (**Figure 2B**).

Mitochondrial function in AKI was further investigated *in vitro* in HK2 cells damaged by cisplatin. Several independent parameters were assessed: mtROS were measured by MitoSOX staining, membrane potential was measured by TMRM staining, and mitochondrial function was evaluated by determining the ATP level and mtDNA copy number. Cisplatin induced excessive mtROS production (**Figure 2C**), massive mitochondrial depolarization (**Figure 2D**) and reductions in ATP production (**Figure 2E**) and the mtDNA copy number (**Figure 2F**) compared with the controls. In contrast, treatment with silybin largely reversed all of these changes. Taken together, these observations indicated that silybin prevented cisplatin-induced mitochondrial dysfunction in HK2 cells.

### Silybin Increases SIRT3 Expression

**FIGURE 3:**
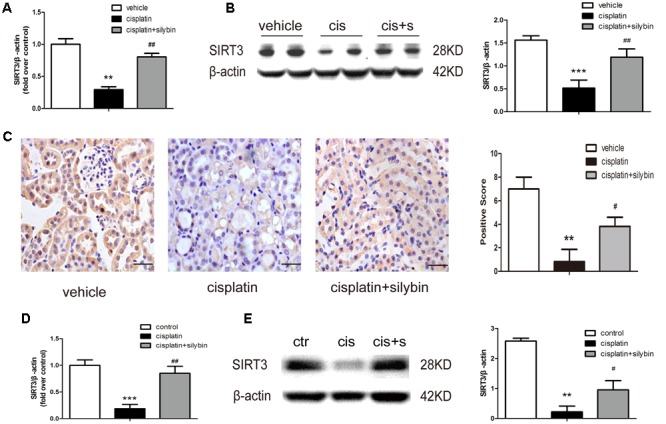
**Silybin can increase the expression of SIRT3 in cisplatin-induced AKI. (A)** Real-time PCR analysis of whole-kidney expression of *SIRT3* mRNA. **(B)** Western blot and densitometric analysis of SIRT3 in the renal tissue of vehicle and cisplatin-treated mice after saline or silybin administration; *n* = 6 mice per group. **(C)** Representative SIRT3 immunohistochemical staining of mouse kidney sections and semi-quantitative positive scoring of SIRT3 staining among different groups. Scale bar, 50 μm. **(D)** Analysis of expression of *SIRT3* transcripts in HK2 cells by RT-PCR. **(E)** Western blot and densitometric analysis of SIRT3 in control and cisplatin-treated HK2 cells in the presence or absence of silybin. Each bar represents the means ± SEM for groups of six mice or three independent cell experiments. ^∗∗^*P* < 0.01, ^∗∗∗^*P* < 0.001 vs. vehicle mice or control cells; #*P* < 0.05, ##*P* < 0.01 vs. mice or cells treated with cisplatin alone. Cis, cisplatin; cis+s, cisplatin+silybin.

A recent study indicated that SIRT3 is a critical mitochondrial protein that protects the kidney against AKI (Perico et al., 2016). PCR and western blotting revealed that SIRT3 expression was clearly decreased in the cisplatin group, while pretreatment with silybin markedly increased SIRT3 expression (**Figures 3A,B**). Immunohistochemical staining also demonstrated that silybin increased SIRT3 expression, especially in tubular epithelial cells, compared with the cisplatin group (**Figure 3C**). Consistently, the mRNA and protein expression of SIRT3 was reduced in cisplatin-treated HK2 cells compared with control cells, while treatment with silybin increased both types of SIRT3 expression (**Figures 3D,E**). Thus, these data indicated that silybin was capable of increasing mitochondrial SIRT3 expression.

### Silybin Increases SIRT3 Expression to Protect against Cisplatin-induced AKI

**FIGURE 4:**
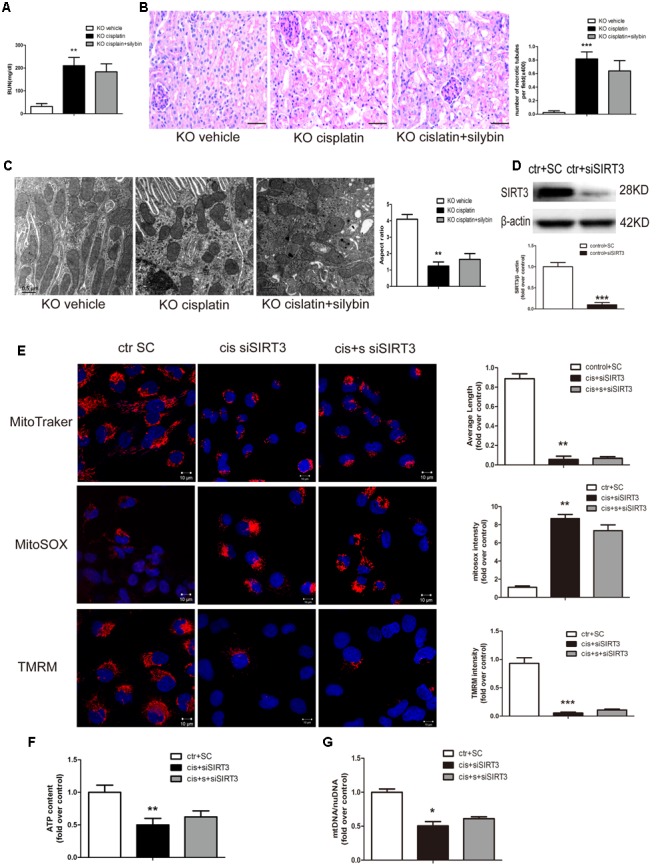
**Silybin protects against cisplatin-induced AKI by enhancing SIRT3 expression. (A)** The graph shows the blood urea nitrogen (BUN) levels of SIRT3 KO mice 72 h after cisplatin injection. **(B)** PAS staining and quantitation of tubular necrosis induced in the vehicle and cisplatin-injected kidneys of SIRT3 KO mice treated with or without silybin. Scale bar, 50 μm. **(C)** Representative TEM and aspect ratio of renal tubular cell ultrastructure in kidney sections of the vehicle and cisplatin-treated SIRT3 KO mice administered saline or silybin. Scale bar, 0.5 μm. **(D)** Western blot and densitometric analysis of SIRT3 protein expression demonstrating the efficiency of transfected SIRT3 siRNA (siSIRT3) compared with scrambled RNA (SC). **(E)** Representative confocal images and quantitative analysis exhibiting the mitochondrial morphology and function of HK2 cells either transfected with scrambled RNA then treated with control medium or transfected with siSIRT3 then exposed to cisplatin alone or with silybin. Mitochondrial morphology was visualized by staining with MitoTracker Deep Red (top), and mtROS and mitochondrial membrane potential were detected using MitoSOX (middle) and TMRM (bottom), respectively. Scale bar, 10 μm. **(F)** ATP levels and **(G)** mtDNA/nDNA ratio were measured in HK2 cells either transfected with scrambled RNA then treated with control medium or transfected with siSIRT3 then treated with cisplatin alone or with silybin. Results are presented as the means ± SEM for groups of six mice or three independent cell experiments. ^∗^*P* < 0.05, ^∗∗^*P* < 0.01, ^∗∗∗^*P* < 0.001 vs. vehicle mice or control cells. KO, knockout; cis, cisplatin; cis+s, cisplatin+silybin; SC, scrambled RNA.

To determine whether silybin protects against cisplatin-induced AKI via activation of SIRT3, we measured the effects of silybin in SIRT3 KO mice. SIRT3 KO mice, along with their WT controls, were injected with cisplatin, either alone or after pretreatment with silybin. After 3 days, significant increases in blood urea nitrogen levels and greater changes in tubular epithelial cells were observed in both the silybin-treated and untreated SIRT3 KO cisplatin-treated mice compared with their WT littermates (**Figures 4A,B**). These results suggested that silybin was unable to protect against cisplatin-induced AKI in the SIRT3 KO mice, whereas it had a protective effect in the WT mice.

Ultrastructural analysis revealed an increased number of swollen and fragmented mitochondria in the SIRT3 KO cisplatin-treated mice compared with the WT cisplatin-treated mice. However, silybin had no effect on elongating the mitochondrial profiles in the SIRT3 KO mice (**Figure 4C**).

To assess mitochondrial function in cisplatin-induced AKI, siRNA was used to downregulate SIRT3 expression. At 48 h after SIRT3 siRNA (siSIRT3) transfection into HK2 cells, SIRT3 protein expression in the siSIRT3-transfected cells was decreased by almost 90% compared with scrambled RNA-transfected cells (**Figure 4D**). Next, the cells were treated with cisplatin in the presence or absence of silybin after transfection with siSIRT3 or were left untreated after transfection with scrambled RNA. The results revealed that silybin could not reverse the changes in mitochondrial morphology, mtROS level, mitochondrial membrane potential (**Figure 4E**), ATP level (**Figure 4F**) or mtDNA copy number (**Figure 4G**), which were all obviously reversed in the silybin-treated WT cells.

In conclusion, these data suggested that the protective effects of silybin in cisplatin-induced AKI involved the activation of SIRT3.

### Silybin Reduces Tubular Cell Apoptosis and Improves Regeneration of these Cells in Kidneys in Cisplatin-induced AKI

**FIGURE 5:**
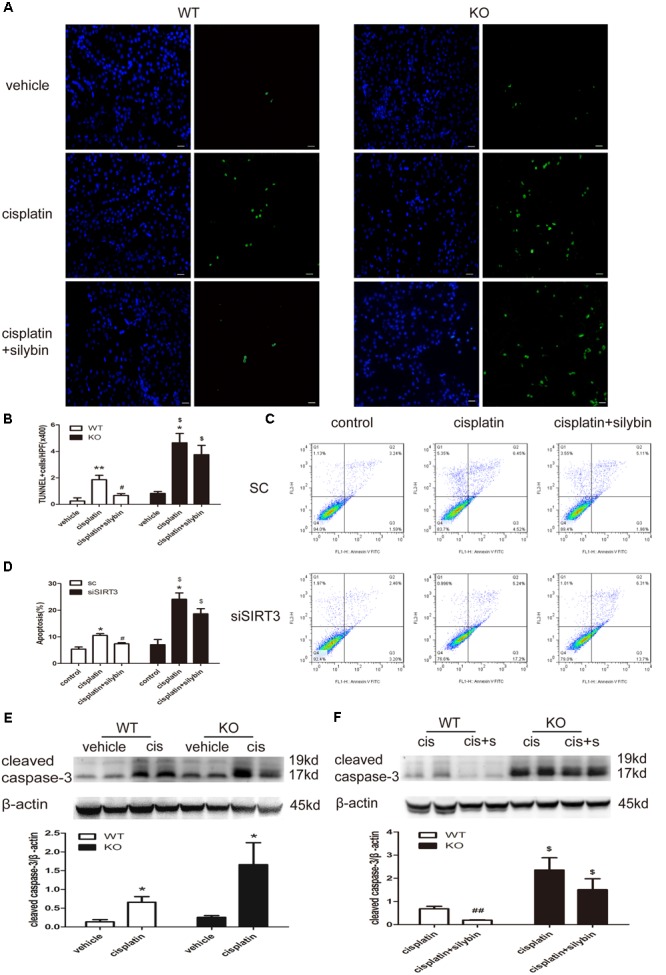
**Silybin reduces tubular cell apoptosis via targeting SIRT3 in kidneys and cells with cisplatin-induced AKI. (A)** Representative micrographs showing a comparison of TUNEL staining in different groups between WT and KO mice. Scale bar, 10 μm. **(B)** Quantitative analysis of the number of TUNEL-positive stained cells per field (×400). **(C)** Representative graphs showing the results of FACS analysis. HK2 cells transfected with scrambled RNA or with siSIRT3 and then exposed to medium or cisplatin in the presence or absence of silybin were stained with annexin-V-FITC and PI to measure cell apoptosis. **(D)** Graph illustrating percentages of cell apoptosis among different groups. **(E,F)** Western blot and densitometric analysis of cleaved caspase-3 protein expression in different groups compared with WT and KO mice. The results are presented as the means ± SEM for groups of six mice or three independent cell experiments. ^∗^*P* < 0.05, ^∗∗^*P* < 0.01 vs. vehicle mice or control cells; #*P* < 0.05, ##*P* < 0.01 vs mice or cells treated with cisplatin alone; $*P* < 0.05 vs. WT mice or scrambled RNA cells from the same group. WT, wild type; KO, knockout; cis, cisplatin; cis+s, cisplatin+silybin; SC, scrambled RNA.

Apoptosis was examined by TUNEL staining *in vivo* (**Figures 5A,B**) and by flow cytometry with Annexin V-FITC and propidium iodide (PI) staining *in vitro* (**Figures 5C,D**). The numbers of apoptotic cells in the cisplatin group, including mice and HK2 cells, were significantly increased compared with the control group, while many fewer apoptotic cells were detected in the silybin group. Moreover, an increased number of apoptotic cells was detected in the SIRT3-deficient cisplatin group compared with the SIRT3-competent cisplatin group; however, silybin pretreatment had no effect on this number in the mice or HK2 cells.

Cleaved caspase-3 expression was also increased in the cisplatin-treated mice compared with the controls for both the WT and SIRT3 KO mice; however, its expression was much higher in the SIRT3 KO cisplatin-treated mice than in the WT cisplatin-treated mice (**Figure 5E**). Pretreatment with silybin greatly decreased cleaved caspase-3 expression in the WT mice but not in the SIRT3 KO mice (**Figure 5F**). These results further support the SIRT3-mediated anti-apoptotic activity of silybin in cisplatin-induced AKI.

**FIGURE 6:**
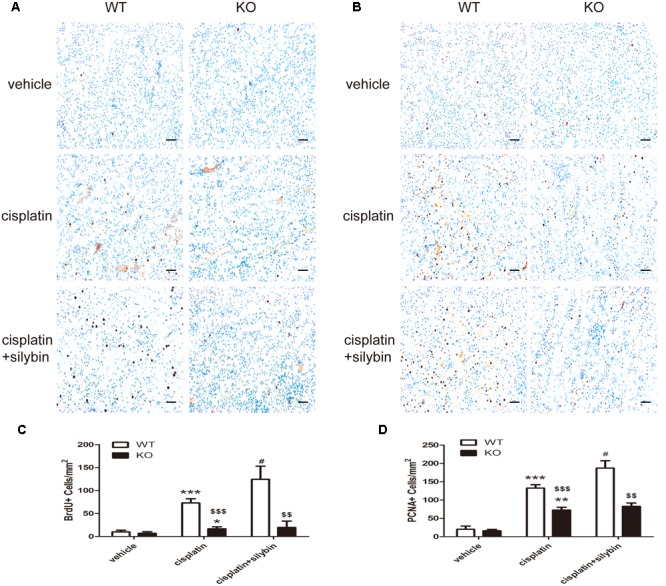
**Silybin treatment promotes tubular epithelial cell regeneration in cisplatin-induced AKI in WT mice, but not in SIRT3 KO mice. (A,C)** Immunohistochemical staining and quantitation of BrdU^+^ cells among different groups compared with WT and KO mice. **(B,D)** Immunohistochemical staining and quantitation of PCNA^+^ cells among different groups compared with WT and KO mice. Scale bar, 10 μm. Each bar represents the means ± SEM for groups of six mice. ^∗^*P* < 0.05, ^∗∗^*P* < 0.01, ^∗∗∗^*P* < 0.001 vs. vehicle mice; #*P* < 0.05 vs mice treated with cisplatin alone; $$*P* < 0.01, $$$ *P* < 0.001 vs. WT mice from the same group. WT, wild type; KO, knockout.

Renal tubular epithelial cell regeneration was assessed by examining BrdU uptake (**Figures 6A,C**) and PCNA staining (**Figures 6B,D**). The results showed that few BrdU-positive (BrdU^+^) and PCNA-positive (PCNA^+^) tubular cells were present in the control group; however, cisplatin injection resulted in substantial increases in the numbers of BrdU^+^ and PCNA^+^ cells in the WT mice but small or no increases in the SIRT3 KO mice. Notably, silybin provided strong protection against cell death in the WT cisplatin-treated mice by promoting cell proliferation, with greater numbers of BrdU^+^ and PCNA^+^ cells, but this effect was not observed in the SIRT3 KO mice. These observations indicate that cisplatin might promote abnormal repair in AKI, and the small number of BrdU^+^ cells detected in the SIRT3 KO cisplatin-treated mouse kidneys suggests that SIRT3 deficiency likely inhibits cell regeneration by preventing the entry of cells into the S phase. Accordingly, treatment with silybin improved cell regeneration in the WT mice with cisplatin-induced AKI, whereas it resulted in few proliferating cells in the SIRT3 KO mice. Therefore, SIRT3 may act as a key regulator of the G1/S transition and in the pharmacologic promotion of AKI repair, as silybin regulated SIRT3 expression to provide strong protection against cisplatin-induced AKI by promoting renal tubular epithelial cell proliferation.

## Discussion

The present study confirmed that SIRT3 plays an important role in regulating mitochondrial function in renal tubular epithelial cells. The results showed that a decrease in SIRT3 expression in renal tubular epithelial cells led to a reduction in mitochondrial bioenergetics in the cisplatin-induced AKI mice. Most importantly, the findings revealed that silybin, the main component of silymarin that has been approved for use in the treatment of liver injury, protected kidneys from cisplatin-induced AKI. Administration of silybin before cisplatin effectively protected mitochondrial function and reduced renal tubular epithelial cell apoptosis. Silybin positively regulated SIRT3 expression in tubular cells and ameliorated renal dysfunction in the cisplatin-induced AKI model mice but had no effect in the SIRT3 KO mice. Thus, enhancing SIRT3 expression with silybin to improve mitochondrial function in tubular cells is a potential strategy for improving the outcomes of renal injury in AKI.

Experimental and clinical studies have shown that silybin provides good protection against various liver diseases, such as alcoholic liver disease, acute and chronic viral hepatitis and toxin-induced liver diseases (Enjalbert et al., 2002; Jacobs et al., 2002; Federico et al., 2006; Rambaldi et al., 2007). The antioxidant and anti-inflammatory properties of silybin are considered to be responsible for its hepatoprotective effects (Ligeret et al., 2008; Trappoliere et al., 2009). Recent reports have demonstrated that silybin is also associated with mitochondrial biogenesis and that it prevents mtROS production by increasing the activities of the superoxide dismutase and peroxidase enzymes in response to liver injury (Detaille et al., 2008; Serviddio et al., 2014). In addition, silybin promotes liver repair by membrane stabilization and immunomodulatory and liver-regenerating mechanisms (Polyak et al., 2007; Gharagozloo et al., 2008; Trouillas et al., 2008). Moreover, it acts as an antifibrogenic agent by reducing the TGF-β-induced synthesis of procollagen type I in human hepatic stellate cells (Trappoliere et al., 2009). However, whether silybin protects renal tubular epithelial cells in cisplatin-induced AKI and the underlying critical role of mitochondria are unknown.

Several lines of evidence indicate that cisplatin nephrotoxicity is mainly associated with mitochondria-generated ROS (Li et al., 2011; Gao et al., 2013). The accumulation of cisplatin in renal epithelial cells can lead to reduced ATP generation, a decline in membrane potential and impaired mitochondrial respiration (Santos et al., 2007; Maimaitiyiming et al., 2013). Our results reinforce the importance of mitochondria as one of the key components of cisplatin nephrotoxicity. We have provided evidence that silybin ameliorates mitochondrial dysfunction in tubular epithelial cells by modulating mitochondrial dynamics and activity. Our data suggest that the administration of silybin leads to a decrease in mtROS and increases in membrane potential, ATP production and mtDNA copy number. Most importantly, this study has unexpectedly revealed that SIRT3 is a mechanistic target of silybin in renal tubular cells in the kidney, through which silybin regulates mitochondrial dynamics and activity.

Sirtuin 3 is mainly located in mitochondria and exhibits global mitochondrial lysine deacetylase activity (Lombard et al., 2007). It participates in the regulation of energy metabolic pathways (Guarente, 2014) through deacetylating various protein in metabolic homeostasis (Martinez-Pastor and Mostoslavsky, 2012; Duan, 2013). In detail, recent studies have highlighted roles of SIRT3 in the control of ATP production, fatty acid oxidation, mitochondrial respiratory chain activity and oxidative stress (Ahn et al., 2008; Bell and Guarente, 2011; Rahman et al., 2014). Moreover, SIRT3 has been linked to the maintenance of mitochondrial morphology through modulation of the fission and fusion processes (Samant et al., 2014; Benigni et al., 2016). In experimental cisplatin-induced AKI, decreased SIRT3 expression is critical for the pathophysiology of tubular injury. SIRT3 prevents mitochondrial fragmentation, leading to decreased tubular cell apoptosis and amelioration of renal damage caused by cisplatin (Morigi et al., 2015). Consistent with these previous findings, our results also indicated that cisplatin reduced SIRT3 expression in the kidney tubules and that SIRT3 deficiency exacerbated AKI. Further, we found that silybin increased SIRT3 expression and ameliorated mitochondrial dysfunction in tubular epithelial cells and kidney injury in AKI mice. However, silybin had no beneficial effects in the SIRT3 KO AKI mice. Our results also demonstrated that silybin reduced apoptosis and increased the proliferation of renal tubular epithelial cells both *in vitro* and in the mouse model. We have provided evidence that silybin might protect the kidneys via the SIRT3 pathway by enhancing renal tubular epithelial cell regeneration, which is crucial for the recovery of renal structure and function after AKI.

Because the induction of apoptosis is the key means by which cisplatin reduces tumor mass, it is unclear whether silybin would counteract the therapeutic effect of cisplatin if it were used as an adjunctive treatment. Fortunately, silybin has been reported to enhance sensitivity to cisplatin therapy in esophageal squamous cell carcinoma, colon adenocarcinoma, and prostate carcinoma (Dhanalakshmi et al., 2003; Leon et al., 2015; Li et al., 2016). However, the different behaviors of silybin in normal and cancerous cells deserve consideration. In particular, a review (Surai, 2015) noted that silybin has been shown to have a protective effect against apoptosis in liver diseases, while it causes apoptosis in cancerous cells. It seems that silybin produces two different effects on normal and cancerous cells. Although the anticancer properties of silybin are well established, these divergent effects remain a mystery. Unraveling this mechanism will represent a significant discovery in the fight against cancer. Thus, the combination of silybin with cisplatin may provide benefits that include not only protection of the kidneys but also antitumor effects.

Collectively, these data suggest that silybin improves mitochondrial function in renal tubular epithelial cells in cisplatin-induced AKI through regulation of SIRT3 expression. Although a large number of effective experimental treatments have been identified for cisplatin-induced kidney injury, none of these treatments are currently used in the clinic. Thus, enhancing SIRT3 expression with silybin to improve mitochondrial function in renal tubular epithelial cells may represent a novel strategy for the prevention of cisplatin nephrotoxicity.

## Ethics Statement

This study was carried out in accordance with the recommendations of the Animal Care Committee of the Sun Yat-sen University of guidelines, the Animal Care Committee of the Sun Yat-sen University of committee. The protocol was approved by the Animal Care Committee of the Sun Yat-sen University of committee.

## Author Contributions

TL, YL, and ZY contributed substantially to the conception of the work. YL, ZY, WL, and WH made the acquisition, analysis, or interpretation of data for the work. TL, YL, and ZY drafted the work for important intellectual content. TL, YL, and ZY made the final approval of the version to be published. JR, XZ, and ZY were took the agreement to be accountable for all aspects of the work in ensuring that questions related to the accuracy or integrity of any part of the work are appropriately investigated and resolved.

## Conflict of Interest Statement

The authors declare that the research was conducted in the absence of any commercial or financial relationships that could be construed as a potential conflict of interest.
